# RISK FACTORS FOR REOPERATION, MORBIDITY, AND MORTALITY IN PATIENTS WITH SMALL BOWEL OBSTRUCTION SUBMITTED TO SURGICAL TREATMENT

**DOI:** 10.1590/0102-672020210002e1654

**Published:** 2022-06-17

**Authors:** Manuel FIGUEROA-GIRALT, Andrés TORREALBA, Tomás GONZALEZ, Paula ALMEIDA, Italo BRAGHETTO, Attila CSENDES

**Affiliations:** 1 Department of Surgery, University Hospital, University of Chile - Santiago - Chile;; 2 Medical Student of University of Chile - Santiago - Chile

**Keywords:** Intestine, Small, Intestinal Obstruction, Risk Factors, Reoperation, Mortality, Intestino Delgado, Obstrução Intestinal, Fatores de Risco, Reoperação, Mortalidade

## Abstract

Small bowel obstruction (SBO) is a frequent cause of emergency department admissions.

**AIM:**

This study aimed to determine risk factors of reoperations, postoperative adverse event, and operative mortality (OM) in patients surgically treated for SBO.

**METHODS:**

This is a retrospective study conducted between 2014 and 2017. Exclusion criteria include gastric outlet obstruction, large bowel obstruction, and incomplete clinical record. STATA version 14 was used for statistical analysis, with p-value <0.05 with 95% confidence interval considered statistically significant.

**RESULTS:**

A total of 218 patients were included, in which 61.9% were women. Notably, 88.5% of patients had previous abdominal surgery. Intestinal resection was needed in 28.4% of patients. Postoperative adverse event was present in 28.4%, reoperation was needed in 9.2% of cases, and a 90-day surgical mortality was 5.9%. Multivariate analysis determined that intestinal resection, >3 days in intensive care unit (ICU), >7 days with nasogastric tube (NGT), pain after postoperative day 3, POAE, and surgical POAE were the risk factors for reoperations, while age, C-reactive protein, intestinal resection, >3 days in ICU, and >7 days with NGT were the risk factors for POAE. OM was determined by >5 days with NGT and POAE.

**CONCLUSIONS:**

Postoperative course is determined mainly for patient’s age, preoperative level of C-reactive protein, necessity of intestinal resection, clinical postoperative variables, and the presence of POAE.

## RESUMO - RACIONAL

A obstrução do intestino delgado (OID) é uma causa frequente de admissões ao Serviço de Emergência.

**OBJETIVO:**

Determinar os fatores de risco de reoperações, eventos adversos pós-operatórios e mortalidade operatória (MO) em pacientes com OID tratados cirurgicamente.

**MÉTODOS:**

Estudo retrospectivo entre 2014 e 2017. Critérios de exclusão: obstrução da saída do estômago, obstrução do intestino grosso e história clínica incompleta. O STATA 14 foi utilizado para análise estatística, considerando significância estatística p<0,05 com IC de 95%.

**RESULTADOS:**

Duzentos e dezoito pacientes foram incluídos, 61,9% mulheres, 88,5% dos pacientes tinham cirurgia abdominal anterior. A ressecção intestinal foi necessária em 28,4% dos pacientes. O evento adverso pós-operatório (EAPO) esteve presente em 28,4%, a reoperação foi necessária em 9,2% dos casos e a mortalidade cirúrgica em 90 dias foi de 5,9%. A análise multivariada determinou que a ressecção intestinal, > 3 dias em UTI, > 7 dias com sonda nasogástrica (SNG), dor após o 3º dia de pós-operatório, EAPO cirúrgico foram fatores de risco para reoperações, enquanto idade, proteína C reativa, ressecção intestinal, > 3 dias em UTI, > 7 dias com SNG foram fatores de risco para EAPO. A MO foi determinada em > 5 dias com SNG e EAPO.

**CONCLUSÕES:**

A evolução pós-operatória é determinada principalmente pela idade do paciente, nível pré-operatório de proteína C reativa, necessidade de ressecção intestinal, variáveis clínicas pós-operatórias e presença de EAPO.

## INTRODUCTION

Small bowel intestinal obstruction (SBO) is a frequent cause of consultation at the emergency unit, representing nearly 20% of all causes of acute abdomen ^7^ ^,^ ^25^. The most frequent etiologies are due to adhesions and internal or external hernia ^3^ ^,^ ^10^. The diagnosis is mainly clinical and supported actually by the computed tomography (CT), which not only improve the accuracy of diagnosis but can also predict small bowel wall vitality ^3^ ^,^ ^7^. The treatment of this disease is mainly surgical; however, nonsurgical treatment has increased in the past years due to the progress of radiological imaging. This conservative management has captured the interest of publications, creating a detriment in the study of complications after surgical approach.

The aim of the present study was to determine risk factors of reoperations, postoperative adverse event (POAE), and surgical mortality (SM) in patients surgically treated for SBO.

## METHODS

### 1. Design

A retrospective analysis of the electronic clinical records of all patients with SBO was submitted to Clinical Hospital University of Chile between January 2014 and December 2017.

Considering the retrospective nature of the study, privacy, and the anonymous analysis of all records, there was no need for Institutional Review Board approval.

### 2. Patients under study

All patients aged 15 years and older with SBO were admitted to our institution and received surgical treatment. All etiologies of SBO were considered.

Exclusion criteria corresponded to patients with gastric outlet obstruction, large bowel obstruction, medical treatment of SBO, and incomplete clinical record.

### 3. Definitions

a. SBO: complete or partial mechanical obstruction from Treitz angle to ileocecal valve.
ii. Diagnose:
1. Clinically if patients had an interruption in their normal bowel transit to gas or feces, plus abdominal pain, abdominal distention, and/or vomits.
2. Radiologically if there was an abnormal small bowel dilatation with a mechanical obstruction.
b. OM: any death occurring from surgery up to postoperative day 90.
c. Zero time for determining prognostic association was surgery.
d. POAEs were classified according to Clavien-Dindo definitions. POAEs were divided into surgical and medical POAE, depending on whether or not they were direct consequence of surgical procedure:
i. Surgical POAE: intestinal perforation, anastomotic leak, surgical-site infection, ileus, and evisceration
ii. Medical POAE: deep vein thrombosis, pulmonary thromboembolism, thrombophlebitis, atelectasis, delirium, and arrhythmia

### 4. Variables studies

a. Preoperative: clinical, laboratory, and tomographic results. Considering that less than 80% of patients had previous clinical records in our hospital, the information that if any anti-adhesion agent were used in the group with previous surgical records was not considered a variable.
i. High inflammatory test was considered with blood cells count >10,000/μl and/or C-reactive protein >5 mg/L.
ii. Acute kidney injury (AKI) was considered when normal urinary (NU)-to-creatinine ratio was >20.
b. Intraoperative: etiology and necessity of intestinal resection.
c. Postoperative: number of days in intensive care unit (ICU), number of days with nasogastric tube (NGT), clinical course, the presence of any POAE, necessity or reoperation, and operative mortality (OM) until 90 days after surgery.

### 5. Statistical analysis

Categorical variables were described in percentages, and parametric variables were expressed as average and standard deviation (SD), according to Shapiro-Wilk test. The Fisher’s exact test, chi-square test, Student’s t-test, and Wilcoxon rank-sum test were performed based on the characteristics and distribution of the variables.

For statistical analysis, STATA^R^ version 14 program was used, with p-value <0.05 considered statistically significant. Univariate and multivariate analyses were performed, calculating the odds ratio (OR) with a 95% confidence interval (95%CI).

The cutoff values for each continuous variable were determined, considering sensitivity, specificity, positive and negative predictive values, and the best area under the receiver operating characteristic curve.

### 6. Follow-up

Follow-up of this study was circumscribed to 90 days after surgery, for proper assessment of OM.

## RESULTS

During the period of this analysis, a total of 431 patients with SBO were admitted. Of these, only 218 (50.6%) cases who were submitted to surgical treatment were included in this study.

The median age was 61.5±18.3 years, and 61.9% were women. Nearly 56% had comorbidities such as hypertension (38.5%), obesity (17.9%), and type 2 diabetes (14.2%). Previous abdominal surgery was reported in 88.5% of the patients, followed by laparotomic approach in 58.4% and laparoscopic in 30.1%.

Table 1 demonstrates the main clinical and radiological findings. The typical symptoms were found in a variable proportion of patients. Abdominal CT with contrast medium showed small bowel dilatation with the absence of gas in the colon in 92.2% of the patients. Free liquid in the abdomen was seen in 37.6% of the patients, and low-contrast enhancement of variable segment of small bowel was demonstrated in 8.3% of the patients. Laboratory results of the presence of inflammation (e.g., elevated white blood cells and increased C-reactive protein) were observed in 70.2% of the cases.

**Table 1 -:** Clinical and laboratory finding.

Variable	Prevalence (%)
Clinical variable	
Abdominal pain	96.3
Absence of gases transit	49.5
Absence of bowel movements	46.8
Vomits	74.7
Peritoneal signs	16.1
Laboratory variable	
High inflammatory tests*	70.2
AKI^	24.8

*: High inflammatory test were considered with blood cells count >10,000/μl and/or C-reactive protein >5 mg/L.

^Acute kidney injury was considered when NU-to-creatinine ratio was >20.

During the surgical procedure, abdominal adhesions were the cause of obstruction (57.3%), followed by internal hernia (16.1%) and abdominal wall hernia (15.1%). Notably, 28.4% underwent intestinal resection due to necrosis.

After the intervention, one-third of the patients had to stay for more than 3 days in ICU. The mean time for the use of NGT was 2.8±4.3 days. Table 2 describes the POAEs from grades 3 to 5, according to the classification of Clavien-Dindo. Reoperation was needed in 9.2% of the patients, due to the following reasons: (1) reobstruction (30%), (2) leak from the enteroenterostomy (35%), and (3) perforation of the small intestine (30%). Mean hospital stay was 12.3±15.9 days (range 1-115). OM was found to be 5.9% up to 90 days after surgery.

**Table 2 -:** Postoperative adverse events according to Clavien-Dindo.

Type of POAE	Prevalence, n=218 (%)
Whole cohort of POAE	62 (28.4%)
Surgical POAE	24 (11% of whole cohort/38.7% of complicated cohort)
CD 1	1 (4.1)
CD 2	1 (4.1)
CD 3	19 (79.1)
CD 4	2 (8.3)
CD 5	1 (4.1)
Medical POAE	59 (27% of whole cohort/97.1% of complicated cohort)
CD 1	5 (8.5)
CD 2	26 (44)
CD 3	14 (23.7)
CD 4	10 (16.9)
CD 5	4 (6.8)

CD: Clavien-Dindo.

Table 3 describes the risk factors for the development of adverse effects after surgery for SBO. Multivariate analysis revealed that 7 of 20 variables were statistically significant contributed factors or independent variables for the presence of postoperative complications: age, age >80 years, C-reactive protein >70 mg/L (normal <5), the presence of internal hernia as the cause of obstruction, necessity of intestinal resection, >3 days in ICU, and >5 days of need of NGT.

**Table 3 -:** Postoperative adverse event risk factors in patients surgically treated for SBO.

Variable	Univariate analysis			Multivariate analysis		
	p	OR	95%CI	p	OR	95%CI
Age	0.03	1.02	1.001-1.03	0.04	1.01	1.001-1.03
Age > 80	0.005	2.69	1.34-5.40	0.009	2.6	1.27-5.29
Sex	0.45					
Comorbidity	0.54					
Days of pain ≥ 4	0.21					
CRP ≥ 70 mg/L	0.021	2.21	1.12-4.35	0.03	2.11	1.05-4.24
CT hypocaptation	0.03	2.77	1.07-7.16	0.13	-	-
Adhesions	0.21					
Internal hernia	0.009	0.27	0.09-0.79	0.11	-	-
Wall hernia	0.31					
Tumor	0.22					
Biliary ileus	0.71					
Intestinal resection	<0.001	3.27	1.75-6.12	<0.001	3.5	1.82-6.71
>3 days ICU	<0.001	14.97	7.36-30.40	<0.001	13.43	6.44-28.01
Days with NGT^	<0.001	1.34	1.21-1.501	<0.001	1.31	1.17-1.47
>5 days with NGT	<0.001	8.36	3.61-19.32			
Reinstallation NGT	0.02	6.75	1.46-12.4	0.27	-	-
Vomits after po day 3	0.15				-	-
Pain after po day 3	0.02	2.49	1.12-5.53	0.55	-	-

CRP: C-reactive protein, lab: laboratory test, AKI: acute renal injury, CT: computed tomography, abd surg: abdominal surgery, ICU: intensive, care unit, NGT: nasogastric tube, po: postoperative, POAE: postoperative adverse event, Surg: surgical, Med: medical.

Table 4 shows the multivariate analysis of risk factors for reoperation after primary surgery for SBO. Out of 33 parameters, 6 were found to be statistically significant: necessity of intestinal resection, >3 days in ICU, the number of days with NGT, abdominal pain after postoperative day 3, the presence of POAE, and the presence of surgical POAE.

**Table 4 -:** Reoperation risk factors in patients surgically treated for SBO.

Variable	Univariate analysis			Multivariate analysis		
	p	OR	95%CI	p	OR	95%CI
Age	0.28					
Age > 80	0.17					
Sex	0.43					
Comorbidity	0.73					
Previous surgery	0.60					
Lower abd surg	0.33					
Laparo surg	0.32					
POAE	0.08					
Abdominal pain	0.54					
Pain > 4 days	0.03	2.66	1.07-6.61	0.23	-	-
Peritoneal sings	0.07					
Inflammatory lab	0.12					
Elevated CRP¨	0.03	2.9	1.13-7.43	0.15	-	-
AKI	0.09					
CT dilatation	0.18					
CT free liquid	0.47					
CT hypocaptation	0.51					
CT neumatosis	0.09					
Adhesions	0.32					
Internal hernia	0.27					
Wall hernia	0.38					
Tumor	0.56					
Biliary ileus	0.90					
Intestinal resection	0.002	4.44	1.75-11.2	0.01	3.6	1.34-9.57
>3 days ICU	<0.001	22.82	5.66-73.45	0.04	5.8	1-05-31.57
Days with NGT^	<0.001	11.51	4.32-30.71	0.038	3.3	1.06-10.11
Reinstallation NGT	0.12					
Vomits after po day 3	0.046	3.87	1.18-12.85	0.23		
Pain after po day 3	<0.001	5.93	2.22-15.92	0.01	4.2	1.31-13.29
POAE	<0.001	19.26	5.73-64.14	0.014	6.02	1.44-25.11
Surg POAE	<0.001	43. 86	14.2-135.2	<0.001	31.3	8.66-113.3
Med POAE	0.005	3.82	1.52-9.54	0.67	-	-

¨≥70 mg/L (sensitivity=30.7%, specificity=83.3%)

^≥7 (sensitivity=55%, specificity=90.4%)

CPR: C-reactive protein, lab: laboratory test, AKI: acute renal injury, CT: computed tomography, laparo surg: laparoscopic surgery, abd surg: abdominal surgery, ICU: intensive, care unit, NGT: nasogastric tube, po: postoperative, POAE: postoperative adverse event, Surg: surgical, Med: medical.

Table 5 demonstrates the risk factors for mortality of patients with SBO submitted to surgical treatment. Out of 27 variables, 7 independent variables had been found to be statistically significant: >5 days with NGT, development of POAE, the presence of surgical POAE, the need for reoperation, the presence of intestinal perforation, the presence of anastomotic leak, and the presence of medical POAE.

**Table 5 -:** Surgical mortality risk factors in patients surgically treated for SBO.

Variable	Univariate analysis			Multivariate analysis		
	p	OR	95%CI	p	OR	95%CI
Age	0.23					
Age > 80 years	0.21					
Sex	0.77					
Comorbidity	0.25					
Days of pain ≥ 4	0.046	3.02	1.02-8.98	0.1	-	-
CRP ≥ 70 mg/L	0.52					
CT hypocaptation	0.29					
Adhesions	0.46					
Internal hernia	0.09					
Wall hernia	0.11					
Tumor	0.19					
Biliary ileus	0.94					
Intestinal resection	0.41					
>3 days ICU	0.008	4.84	1.51-15.37	0.23	-	-
Days with NGT^	0.032	1.09	1.002-1.19	0.47	-	-
>5 days with NGT	<0.001	6.46	2.09-20.02	0.02	4.4	1.24-15.64
Reinstallation NGT	0.35					
Vomits after po day 3	0.64					
Pain after po day 3	0.48					
POAE	<0.001	9.81	2.78-34.23	0.015	7.1	1.46-34.58
Surg POAE	0.001	6.11	1.91-19.76	0.005	6.1	1.73-21.16
Reoperation	<0.001	11.7	3.61-38.16	0.04	5.8	1.01-33.51
Intestinal obstruction	0.21					
Perforation	0.003	20.2	4.11-100.4	0.03	8.3	1.21-56.95
Anastomotic leak	<0.001	22.3	5.22-96.81	0.003	16.9	2.67-106.6
Med POAE	0.007	4.83	1.58-14.68	0.042	3.8	1.04-13.62

CRP: C-reactive protein, lab: laboratory test, CT: computed tomography, abd surg: abdominal surgery, ICU: intensive, care unit, NGT: nasogastric tube, po: postoperative, POAE: postoperative adverse event, Surg: surgical, Med: medical,

## DISCUSSION

The results of this study suggest the following:

1) There is a change in the causes of SBO, with an increase of internal hernias as the second cause.
2) There are several risk factors associated with the complications after surgical treatment of SBO.
3) Several variables can be identified as risk factors for reoperation and OM.

The etiology of SBO has remained similar through decades (2,3,10), with the presence of abdominal adhesions being responsible for nearly 93% of the patients. Approximately 30% of SBO occur in the first year after operation and 3% debut with SBO 10 years after initial surgical procedure ^18^. In this study, the presence of internal hernias appears as the second cause of etiology, over abdominal wall hernias; this might be probably due to the high incidence of bariatric and oncological procedures with Roux-en-Y reconstruction ^14^. The impact of laparoscopic surgery in cumulative incidence of SBO remains unknown; however, with the reduction of adhesions and incisional hernias after minimally invasive surgery, it is probable that internal hernias as cause of SBO may continue to increase.

The natural history following adhesiolysis is not completely understood. There is evidence suggesting that SBO may recur up to 32% of patients, with a cumulative incidence of recurrence of 3.7% at 1 year and 5.8% at 5 years.

The dilemma of surgical versus medical treatment has been evaluated in several studies ^1^ ^,^ ^3^ ^,^ ^10^ ^,^ ^19^ ^,^ ^26^. In one study, up to 60-80% of patients have been resolved conservatively ^3^; but in this study, only 50% were operated on, which is in contrast to our previous study in which nearly 75% of patients were submitted to surgical approach ^10^. However, the shorter the hospital stay with conservative management, the higher the chance and the shorter the time to recurrence ^1^ ^,^ ^10^ ^,^ ^19^ ^,^ ^26^. Besides, the risk of small bowel ischemia and necessity of intestinal resection in cases with failed medical treatment can be as high as 75% ^10^.

Clinical and laboratory examinations have been the classical way to diagnose SBO. The widespread use of triphasic CT has enhanced greatly the diagnosis of complete or incomplete obstruction. To predict better the patient’s evolution, international reports have focused on the utility of noninvasive tests such as oral water soluble contrast (OWSC), which has a strong predictive value in defining conservative treatment, with only 3% of surgical necessity in patients with incomplete obstruction ^1^ ^,^ ^8^ ^,^ ^19^. The impact of OWSC in malignant obstruction has been evaluated in a recent systematic review ^23^, thus finding insufficient evidence to determine the need of conservative treatment with this method.

In terms of surgical approach, studies suggested that laparoscopic approach is feasible in nearly 60% of the patients, but with careful selection ^22^ ^,^ ^24^. Several publications ^9^ ^,^ ^15^ ^,^ ^20^ suggested that laparoscopic treatment could be better than laparotomic approach in terms of complications (OR 0.7), incisional complications (OR 0.22), and 30-day mortality (OR 0.55). In the present series, the rate of complications and mortality were 28.4% and 5.9%, respectively, as compared to other publications ^15^ ^,^ ^16^ ^,^ ^21^ ^,^ ^22^.

Risk factors for complications after surgery for SBO have been previously reported. Suter et al concluded that there were no risk factors for intraoperative complications, but accidental bowel perforation (p<0.008) and the need for conversion (p<0.009) were independent variables for an increased risk of POAE (13). This is in contrast with the conclusion suggested by Dindo ^11^, in which these variables plus ASA >2 were significant in univariate analysis, but in multivariate analysis only conversion due to intraoperative complication was an independent factor for POAE (OR:3.97). In contrast, Duron found that surgical adverse effects were associated with the number of obstructed structures (OR:8.3), nonresected intestinal wall injury (OR:5.3), and intestinal necrosis (OR:5.6). Our results demonstrated that age, especially >80 years, was an independent factor, as mentioned in a study by Chang ^6^. Besides, the need for intestinal resection and prolonged stay in ICU were also independent variables. The finding that C-reactive protein >70 was an important risk factor has not been evaluated in other studies.

Although reoperation is a predominant factor for increased morbidity and mortality, only few studies have reported this fact. Our study had a value of 9.2%, as compared to other studies ^5^ ^,^ ^10^ ^,^ ^17^ ^,^ ^22^. This study is the first to report on the multivariate analysis of reoperation risk factors in those who were surgically treated.

The actual OM of our study was 5.9%, similar to other series ^10^ ^,^ ^12^ ^,^ ^17^. However, this study only included surgically treated patients, who usually have a worst condition compared to medically treated cases. Other publications have shown that age, preoperative functional status, ASA stage, medical complications, mixed mechanism of obstruction, intestinal resection, malignant etiology, and reoperation influence OM significantly ^6^ ^,^ ^7^ ^,^ ^12^. In our series, we found that only postoperative variables determine OM.

There are several limitations in this study: (1) it is a retrospective analysis with all the bias; (2) the laparoscopic group represent a small fraction of the entire cohort and therefore were excluded from the analysis; and (3) there is no longer follow-up in order to determine recurrence of SBO.

## CONCLUSION

The findings of this study suggest that postoperative course of a patient with SBO submitted to surgical treatment is determined by patient’s age, preoperative C-reactive protein level, necessity of intestinal resection, and the presence of POAEs.
